# Origin and evolution of SARS-CoV-2

**DOI:** 10.1140/epjp/s13360-023-03719-6

**Published:** 2023-02-16

**Authors:** Isabel Pagani, Silvia Ghezzi, Simone Alberti, Guido Poli, Elisa Vicenzi

**Affiliations:** 1 Viral Pathogenesis and Biosafety Unit, Division of Immunology, Transplantation, and Infectious Diseases, San Raffaele Scientific Institute, Via Olgettina, 58, Milan, Italy; 2 Human Immuno-Virology (H.I.V.) Unit, Division of Immunology, Transplantation, and Infectious Diseases, San Raffaele Scientific Institute, Via Olgettina, 58, Milan, Italy; 3 Vita-Salute San Raffaele University School of Medicine, Via Olgettina, 58, Milan, Italy

## Abstract

SARS-CoV-2 is a novel coronavirus that emerged in China at the end of 2019 causing the severe disease known as coronavirus disease 2019 (COVID-19). SARS-CoV-2, as to the previously highly pathogenic human coronaviruses named SARS-CoV, the etiological agent of severe acute respiratory syndrome (SARS), has a zoonotic origin, although SARS-CoV-2 precise chain of animal-to-human transmission remains undefined. Unlike the 2002–2003 pandemic caused by SARS-CoV whose extinction from the human population was achieved in eight months, SARS-CoV-2 has been spreading globally in an immunologically naïve population in an unprecedented manner. The efficient infection and replication of SARS-CoV-2 has resulted in the emergence of viral variants that have become predominant posing concerns about their containment as they are more infectious with variable pathogenicity in respect to the original virus. Although vaccine availability is limiting severe disease and death caused by SARS-CoV-2 infection, its extinction is far to be close and predictable. In this regard, the emersion of the Omicron viral variant in November 2021 was characterized by humoral immune escape and it has reinforced the importance of the global monitoring of SARS-CoV-2 evolution. Given the importance of the SARS-CoV-2 zoonotic origin, it will also be crucial to monitor the animal-human interface to be better prepared to cope with future infections of pandemic potential.

## Introduction

Since the beginning of 2020, our lives have been profoundly changed by the emergence of the worldwide SARS-CoV-2 spreading [1, 2] that can cause a disease known as Coronavirus disease 2019 (COVID-19) involving pneumonia and multiorgan failure [3, 4] with a significant fatality rate that varies among countries and estimated to be approximately 1–2% of all infected individuals who have not been vaccinated [5]. SARS-CoV-2 belongs to the large family of coronaviruses that are enveloped viruses, characterized by a single-stranded positive RNA genome of approximately 30,000 nucleotides in size, the largest known genome for RNA viruses [6]. Despite coronaviruses are widely present in the animal world, in humans, seven pathogenic coronaviruses have been identified in the last 20 years [7]. Out of these, three have raised special concern as they are highly pathogenic causing severe pneumonia and death. The first human coronavirus of public health concern was SARS-CoV that emerged in the Guangdong Province of China in 2002 [8, 9]. Approximately 8,000 cases and 700 deaths were reported during the virus’s eight-month circulation in 29 countries and five continents, but, by the end of July 2003, the virus disappeared from the human population, thanks to the containment measures [10, 11].

In 2012, another coronavirus, named Middle East respiratory syndrome (MERS)-CoV, emerged in Saudi Arabia [12, 13]. Unlike SARS-CoV, this virus is still present in the human population, albeit infections are sporadic and limited to the Arabic peninsula with only 12 cases reported in 2021 (https://www.ecdc.europa.eu/en/publications-data/geographical-distribution-confirmed-mers-cov-cases-1-january-2021-2-august-2021).

At the end of 2019, a novel SARS-CoV-2 emerged in China. In contrast to the previous human coronaviruses, it has been spreading very efficiently by infecting more than 670 million individuals worldwide causing more than 6.8 million deaths at the time of this report (https://coronavirus.jhu.edu/map.html). Although both SARS-CoV and SARS-CoV-2 are airborne viruses [14], SARS-CoV is transmitted during the symptomatic phase of the infection whereas SARS-CoV-2 can be transmitted prior to the onset of symptoms and from asymptomatic individuals [15], who represent approximately 40–45% of all infected individuals [16], as efficiently as symptomatic individuals. This SARS-CoV-2 peculiarity poses a challenge for the public health systems to contain the spreading of the infection. Importantly, however, effective vaccines have been developed in a record time (11 months) to provide global containment of this pandemic [17, 18].

## Origin of SARS-CoV-2

**Fig. 1:**
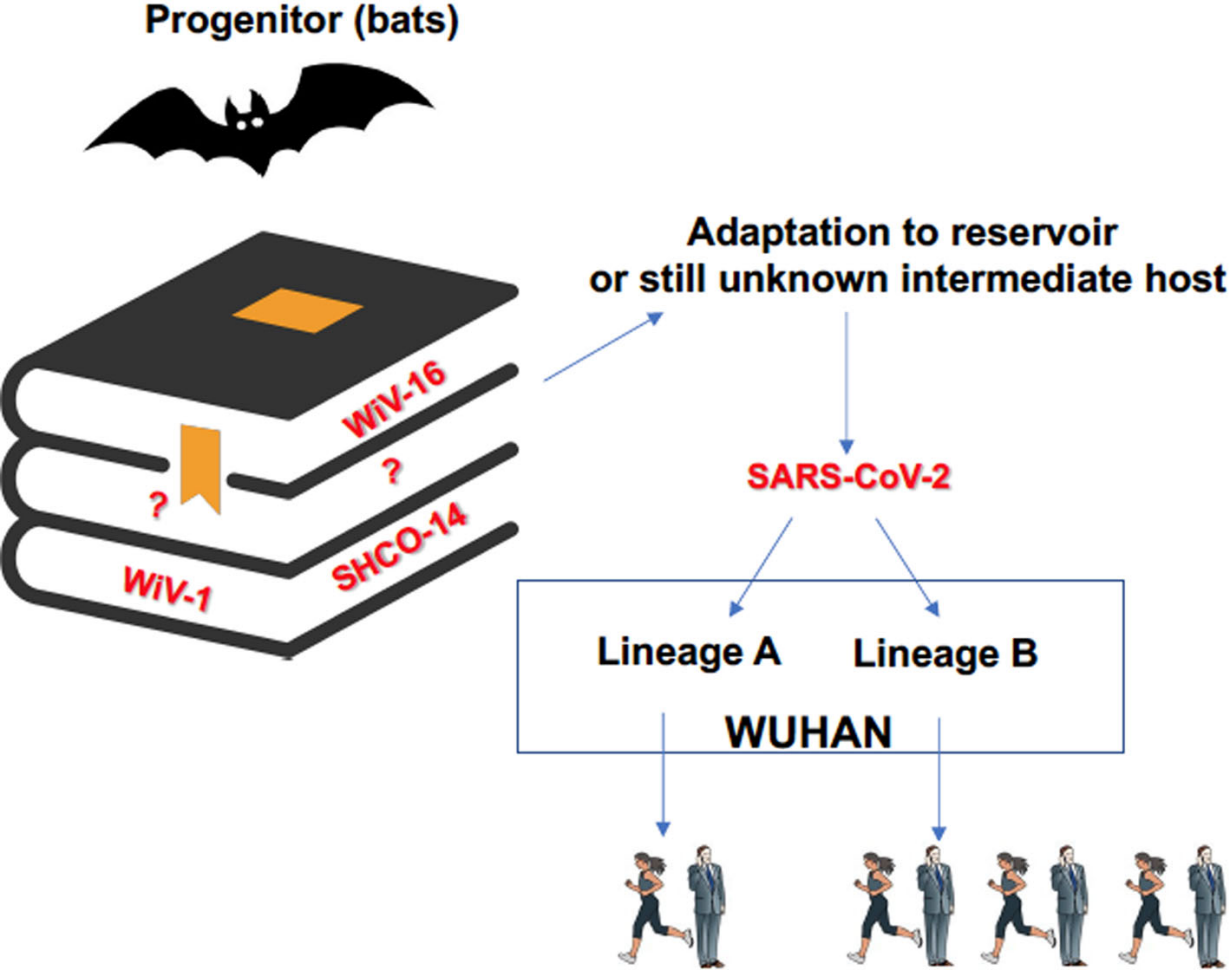
Scenario of SARS-CoV-2 natural origin

All previous highly pathogenic human coronaviruses have had a zoonotic origin. SARS-CoV, which shares *ca*. 79% homology at the nucleotide sequence level with SARS-CoV-2, was linked to live animals sold at the markets in Foshan, Guangdong Province, China [19, 20]. Viral isolates sharing 99.8% homology with SARS-CoV were obtained from a few Himalayan palm civets and one raccoon dog and animal traders without a SARS diagnosis were found to have high levels of antibodies (Abs) against SARS-CoV in late 2002 [21]. However, several years of research surveying wild animals revealed that bats are infected with the ancestral viruses of SARS including SARS-CoV-2 and, thus, bats serve as a main reservoir of many SARS-like viruses [22]. The first evidence came from the isolation of the closest known relatives to SARS-CoV but also SARS-CoV-2 from horseshoe (*Rhinolophus*) bats that colonize caves in the Yunnan Province of mainland China [1, 23]. However, the notable geographic gap between the Yunnan caves and the location of the first emergence of SARS-CoV and SARS-CoV-2 highlights the difficulty of precisely trace the virus’s movements and the importance to sampling other regions and countries. In this regard, viruses more closely related to SARS-CoV-2 than those from the Yunnan region [24] have been documented in bats but also in pangolins from multiple locations in South-East Asia, including China, Thailand, Cambodia and Japan [25–27]. As a significant evolution gap exists between SARS-CoV-2 and the closest-related animal viruses, it is unlikely that SARS-CoV-2 spilled over directly from bats to humans. Likely, the spillover event(s) occurred in the context of locations where high-density population is in contact with live animals susceptible to SARS-CoV-2 infection and sold alive for food in the Wuhan markets that were the early and major epicenters of SARS-CoV-2. However, these highly related viruses were not identified in the animals present in the Wuhan markets during the initial phase of COVID-19 pandemic (Fig. 1). This gap has fostered the speculation of a virus escape from the Wuhan Institute of Virology (WIV) where the research on SARS-related viruses were intensified after the 2002–2003 SARS-CoV global spread. In this regard, the analysis of the early Wuhan sequences identified two independent lineages of SARS-CoV-2 that were simultaneously distributed at different Wuhan wildlife markets and likely transmitted by a still unidentified wild-caught or farmed animals sold in the markets [28]. To fully demonstrate the natural origin of SARS-CoV-2, it will be fundamental to extensively survey the presence of coronaviruses in wild animals and studying their evolution and ecology.

Another important aspect of SARS-CoV-2 ecology is the cross-species transmission [29]. Indeed, SARS-CoV-2 can be transmitted from human-to-animal as documented in farmed minks [30], dogs and cats [31] as well as in lions and tigers in zoos [32]. Upon animal infection, the human virus may evolve and adapt to the new host. Viral evolution occurred in minks in the Netherlands and Denmark [33] where the human-to-animal transfer resulted in the introduction of an adaptive mutation in the receptor binding domain (RBD) of the viral Spike protein. A tyrosine was replaced by phenylalanine at position 453 (Y453F) causing an increase in the Spike protein affinity for the viral entry receptor angiotensin converting enzyme-2 (ACE2) [33]. Furthermore, infection of animals with human SARS-CoV-2 may pose a serious problem as recombination events may occur among animal and human coronaviruses [34] to generate novel hybrid viruses with pandemic potential if they spillover in humans with no or partial immunity.

## SARS-CoV-2 evolution

**Fig. 2:**
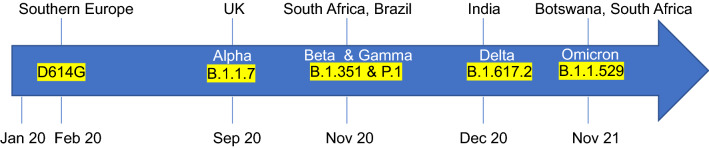
Significant SARS-CoV-2 variants and countries and dates of first detection

SARS-CoV-2, similarly to all RNA viruses, is prone to accumulating mutations in its genome during each cycle of replication. However, coronaviruses encode a 3′-to-5′-exonuclease that allows high fidelity replication by the viral RNA-dependent-RNA polymerase [35, 36]. This proofreading activity is thought to be necessary for maintaining the integrity of a long viral RNA genome and limiting the error rate imposed by viral RNA polymerases [37]. However, SARS-CoV-2 replicates very efficiently in infected hosts as demonstrated by the elevated viral loads measured in the nasopharyngeal swabs [38]. From December 2019 to November 2020, the evolution rate of SARS-CoV-2 suggests that its genome acquires approximately 2 mutations per month [39, 40]. However, over the course of the pandemic, several mutations have emerged, particularly in the Spike protein, implying that SARS-CoV-2 evolution rate is higher than predicted. By late February 2020 a single non-synonymous substitution of an aspartic acid into glycine was detected in the Spike protein at position 614 (D614G) [41] (Fig. 2).

Firstly, a minority of the sequences (5%) available in the data base, particularly in southern Europe, had the 614G, but with the spreading of the virus, this variant become the most prevalent genotype worldwide [41]. Importantly, in vitro studies have demonstrated that the glycine residue in position 614 confers a higher efficiency in viral entry and replication than that of Spikes with the aspartic acid residue [41, 42]. Furthermore, competition studies with original and mutant viruses generated by reverse genetics clearly demonstrated the higher fitness of the virus with the 614G than 614D [42]. These results were confirmed in vivo in a model of SARS-CoV-2 infection in Syrian hamsters [42]. Despite the increased infectivity and viral load in infected people, increased COVID-19 disease severity was not observed in association with D614G mutation, although infection rates were extended to younger patients [43]. Importantly, the D614G variant remained sensitive to the neutralizing activity of sera from individuals who have received vaccine preparation based on the original Wuhan Spike sequence and human monoclonal Abs targeting the Spike protein [44].

With the unfolding pandemic, an unprecedented amount of full genome sequencing data was made available via the Global Initiative on Sharing All Influenza Data (GISAID—https://www.gisaid.org/). In September 2020, the analysis of the continuous increase in genomic data highlighted the emergence of sequences characterized by a higher number of mutations relative to the previous circulating SARS-CoV-2. A new lineage defined as B.1.1.7 was identified in the United Kingdom (UK). This new variant was characterized by 14 non-synonymous and 6 synonymous substitutions and 3 deletions along the entire genome that mapped to a single monophyletic branch of the phylogenetic tree [43, 45]. Eight of these mutations, including substitutions and deletions, were positioned in the Spike protein that interacts with the cellular receptor to mediate viral entry into target cells [46–49]. Of note is the mutation in position 501 of the Spike protein where an asparagine residue is changed into a tyrosine residue (N501Y). This position is the principal contact residue in the RBD and it was shown to increase the Spike affinity for ACE2 by sixfold [50, 51].

The emergence of the B.1.1.7 variant has paved the way for more attention to the SARS-CoV-2 evolution as, by November 2020, the virus had started to mutate more drastically. Variants of concern (VOCs) with mutations of note in the Spike protein started to emerge, being characterized by increased transmissibility and changes in antigenicity, thus, raising the threat level to the public health. The three most notable VOCs came from South Africa (B.1.351) [52], Brazil (P.1) [53] and India (B.1.617.2) [54]. However, an abundance of variants of interest (VOIs) including B.1.525 (Nigeria), B.1.526 (New York), B.1.427/B.1.429 (California), B.1.258 (Scotland), and A.23.1 (Liverpool) have emerged which collectively now account for more than 90% of the sequenced viruses [55]. Given the complexity of the multiple variants, recently, the WHO has changed the nomenclature of VOCs and VOIs with the letters of the Greek alphabet re-naming B.1.1.7 as Delta, B.1.351 as Beta, P.1 as Gamma and B.1.617.2 as Delta (Fig. 2) whereas VOI were renamed for B.1.525 as Eta, B.1.526 as Iota, B.1.617.1 as Kappa, C.37 as Lambda and B.1.621 as Mu.

Worth noting, however, is the Delta variant that emerged in India at the end of 2020 [56] and since then it has been spreading in and outside India to become the predominant variant in Europe and worldwide [57, 58]. Epidemiological data indicate that the Delta variant is 40 to 60% more transmissible than Delta and, importantly, Delta variant replicates more efficiently than the previous variants as demonstrated by the viral loads in the nasopharyngeal swab reported to be *ca*. 1000-fold higher than that of the infection with the previous variants [59]. The higher efficiency of Delta to infect and replicate is explained by a set of three mutations in the Spike RBD. Firstly, a lysine to asparagine mutation at position 417 is present in some, but not all sequences. This mutation has been associated with conformational changes in the Spike protein that may result in immune escape [60]. The second mutation, a leucine to arginine change at position 452 increases the affinity for ACE2 receptor and the third, a threonine to lysine substitution at position 478, is common to the B.1.1.519 lineage, and it has been also predicted to increase RBD/ACE2 binding affinity and enable immune escape [54]. In addition, a proline to arginine substitution is present at position 681 adjacent to the furin cleavage site of the Spike protein that increases the efficiency of its cleavage to mediate viral fusion and initiate infection.

Unexpectedly, however, a new VOC, named B.1.1.529, was detected for the first time in Botswana and South Africa in November 2021 and later renamed Omicron [61]. This VOC has immediately raised concerns due to its high number of mutations, particularly in the Spike protein. Multiple changes have been detected within the two immunogenic regions in S1 (N-terminal domain (NTD and RBD) including a 3-amino acid insertion and an accumulation of mutations in proximity of the furin cleavage site including the combination of N679K and P681H mutations. Overall, these mutations render this virus more infectious than previous variants [62] with a short doubling time [63]. However, in vivo experimental infection of human ACE2 transgenic mice and Syrian hamsters has shown that Omicron causes attenuated disease as compared with D614G and Beta variants due to an impaired capacity to spread in the lower respiratory tract [64]. Importantly, unlike previous VOC, the Omicron mutations have lowered the neutralization activity of the Abs raised by vaccination by *ca.* 22-fold [65] rendering the human population more susceptible to infection. The rapid spread of Omicron has replaced the previous Delta variant in a few weeks worldwide and signs of viral evolution have been detected as determined by the emergence of sub-variants [66]. After the initial BA.1 emersion, the BA.2 sub-lineage has increased in many areas globally in a few weeks whereas the BA.3 has not taken off yet globally. While BA.2 and BA.3 share many of the changes present in BA.1, they are also characterized by unique mutations that define distinct groups in the phylogenetic analysis of multiple sequences [61]. Thus, the rapid spread, evolution and immune evasion of Omicron with its sub-variants continue to represent a concern for its containment.

## Concluding remarks

SARS-CoV-2 is a novel coronavirus with a likely zoonotic origin albeit the precise spill over event(s) has not been elucidated. Animal-to-human transmission was documented for other highly pathogenic coronaviruses whereas human-to-animal transmission had been documented for SARS-CoV-2. This interspecies transmission has raised concern as coronaviruses can easily adapt to their host to become more infectious and can recombine with animal coronavirus generating novel coronaviruses with pandemic potential.

Regarding SARS-CoV-2 adaptation in humans, several variants named as VOCs and VOIs have emerged in a relatively short period of time. The mechanism under which VOCs and VOIs have emerged is unclear [67]. There is convincing evidence that the variants likely develop in chronic infection of immunocompromised hosts receiving convalescent sera [68–70]. In these conditions, a suboptimal immune response to the virus is a perfect milieu for the selection of variants characterized by higher efficiency of viral replication and immune evasion. The availability of an unprecedented sequence data set and their analysis have supported the presence of signatures of positive selection dependent on the immune response to the virus. VOCs and VOIs are characterized by several defining and converging mutations that have emerged in a relatively short period suggesting that the evolution rate of SARS-CoV-2 is several folds higher than at the beginning of the pandemic when the evolution rate was estimated to be two mutations per month. This continuous evolution that reminds the antigenic drift observed in Influenza A virus [29] is a topic of concern for the effectiveness of the current vaccines. A rapid and broad immunization campaign, particularly focusing on the poor regions of the world will be pivotal to contain the current SARS-CoV-2 pandemic.
